# Effectiveness of electrical stimulation on rehabilitation after ligament and meniscal injuries: a systematic review

**DOI:** 10.1590/S1516-31802011000600008

**Published:** 2011-12-01

**Authors:** Aline Mizusaki Imoto, Stella Peccin, Gustavo Jerônimo Melo Almeida, Humberto Saconato, Álvaro Nagib Atallah

**Affiliations:** I PhD. Postgraduate Program on Internal Medicine and Therapeutics, Universidade Federal de São Paulo (Unifesp), São Paulo, Brazil.; II PhD. Supervising Professor of the Postgraduate Program on Internal Medicine and Therapeutics, Universidade Federal de São Paulo (Unifesp), São Paulo; and Head of the Department of Movement Sciences, Universidade Federal de São Paulo, Campus Baixada Santista, São Paulo, Brazil.; III PhD. Physiotherapist, Department of Physical Therapy, University of Pittsburgh, Pittsburgh, United States.; IV PhD, Adjunct Professor, Department of Clinical Medicine, Universidade Federal do Rio Grande do Norte (UFRN), Natal, Rio Grande do Norte, Brazil.; V PhD. Titular Professor, Department of Internal Medicine, Universidade Federal de São Paulo (Unifesp), São Paulo, Brazil.

**Keywords:** Electric stimulation therapy, Rehabilitation, Knee joint, Knee injuries, Physical therapy modalities, Terapia por estimulação elétrica, Reabilitação, Articulação do joelho, Traumatismos do joelho, Modalidades de fisioterapia

## Abstract

**CONTEXT AND OBJECTIVE:**

Electrical stimulation (ES) is widely used to strengthen muscles following ligament and meniscal injuries. The aim of this study was to evaluate the effectiveness of ES for rehabilitation after soft tissue injuries of the knee treated surgically or conservatively.

**DESIGN AND SETTING:**

Systematic review at the Brazilian Cochrane Center.

**METHODS:**

We searched the Cochrane Central Register of Controlled Trials (2010, Issue 12), Medline (Medical Analysis and Retrieval System Online) via PubMed (1966 to December 2010), Embase (Excerpta Medica database, 1980 to December 2010), Lilacs (Literatura Latino-Americana e do Caribe em Ciências da Saúde, 1982 to December 2010), and PEDro (Physiotherapy Evidence Database, 1929 to December 2010). The studies included were randomized controlled trials using ES to increase muscle strength for rehabilitation of patients with soft tissue injuries of the knee. Two authors independently evaluated studies for inclusion and performed data extraction and methodological quality assessment.

**RESULTS:**

Seventeen studies evaluating ES after anterior cruciate ligament reconstruction and two studies evaluating ES after meniscectomy were included. There was a statistically significant improvement in quadriceps strength through ES (mean difference, MD: −32.7; 95% confidence interval, CI: −39.92 to −25.48; n = 56) and in functional outcomes (MD −7; −12.78 to −1.22; n = 43) six to eight weeks after surgical reconstruction of the anterior cruciate ligament.

**CONCLUSION:**

There is evidence that ES coupled with conventional rehabilitation exercises may be effective in improving muscle strength and function two months after surgery.

## RESUMO

**CONTEXTO E OBJETIVO:**

A estimulação elétrica (ES) é amplamente utilizada para fortalecimento muscular após lesões ligamentares ou meniscais do joelho. O objetivo deste estudo foi avaliar a efetividade da ES na reabilitação de lesões de tecidos moles do joelho tratadas de forma cirúrgica ou conservadora.

**TIPO DE ESTUDO E LOCAL:**

Revisão sistemática no Centro Cochrane do Brasil.

**MÉTODOS:**

Realizamos uma busca no Cochrane Central Register of Controlled Trials (2010, Issue 12), Medline (Medical Analysis and Retrieval System Online) via PubMed (1966 até dezembro 2010), Embase (Excerpta Medica Database, de 1980 até dezembro 2010), Lilacs (Literatura Latino-Americana e do Caribe em Ciências da Saúde, de 1982 até dezembro de 2010), and PEDro (Physiotherapy Evidence Database, de 1929 até dezembro de 2010). Os estudos incluídos foram ensaios clínicos randomizados que utilizaram a ES com o objetivo de aumento de força muscular na reabilitação de pacientes com lesões de tecidos moles. Dois autores avaliaram os estudos para inclusão de forma independente e realizaram a extração de dados e avaliação da qualidade metodológica.

**RESULTADOS:**

Dezessete estudos incluídos utilizaram a ES após a reconstrução do ligamento cruzado anterior e dois estudos após meniscectomia. Houve melhora estatisticamente significante na força do quadríceps através da ES (diferença média, MD −32.7; 95% intervalo de confiança, IC −39.92 to −25.48; n = 56) e nos desfechos funcionais (MD −7; −12.78 to −1.22; n = 43), seis a oito semanas após cirurgia de reconstrução do ligamento cruzado anterior.

**CONCLUSÃO:**

Há evidências de que a ES combinada a exercícios de reabilitação convencional pode ser efetiva na melhora da força muscular e função dois meses após cirurgia.

## INTRODUCTION

Knee injuries can be considered to be a modern epidemic.^1^ They are more common among people between 10 and 29 years of age and occur more often among men (57%). Soft tissue injuries of the knee most frequently involve the menisci or knee ligaments. The incidences of these injuries are 0.3 and 0.7 per 1000 individuals/year, respectively.^1^ The anterior cruciate ligament (ACL) is the ligament most frequently damaged, and the medial meniscus is the most frequently damaged meniscus. One study investigating associations between ACL and meniscal injury^2^ found that 86% of the patients with ACL insufficiency had an associated meniscal injury, while 58% presented this association in another study.^3^

In general, knee soft tissue injuries occur when the knee twists while bearing weight. The injury severity depends on the excess movement and force on the knee joint.

Muscle wasting and weakness are well-known effects following joint surgery and immobilization. Improvement of muscle strength is a major goal to be achieved early during rehabilitation.^4^ For example, atrophy and weakness quickly develop in the quadriceps muscle after ACL reconstructive surgery.^5,6^ Five to six weeks of immobilization following serious ligament injury or surgery induces significant changes in type 1 muscle fibers in the quadriceps^7,8^ and 60% to 80% reduction in isometric quadriceps strength.^9^ Muscle strength is important for recovering physical function, and compromised thigh muscle strength has been shown to be associated with abnormalities of walking velocity, stride length and pace.^10^

Avoidance of isolated use of the knee extensor muscle, in order to protect the newly made ligament, and time^11,12^ are the two main requirements in the rehabilitation process. Rehabilitation may include exercises and electrostimulation (ES) to improve quadriceps strength.^13,14^ ES is recommended as an adjunct treatment for quadriceps strengthening after anterior cruciate ligament reconstruction and has also been shown to improve quadriceps femoral torque produced after ligament knee surgery.^15,16^ The most frequently used currents are: alternating current (2500 Hz) and pulsed biphasic asymmetric rectangular current. The choice of electrical current must be based on the capacity of electrical stimulation to produce an effective contraction.^17^ ES is widely used to strengthen muscles after soft tissue injuries of the knee such as ligament and meniscal injuries, and after surgery to treat these conditions, such as anterior cruciate ligament reconstruction. It is therefore important to conduct a systematic review to assess the effectiveness of electrical stimulation for improving the strength of muscles following soft tissue injuries of the knee, treated conservatively or surgically.

## OBJECTIVES

To assess the effectiveness of ES for rehabilitation after soft tissue injuries of the knee (anterior cruciate, posterior cruciate, lateral and medial ligament and meniscal injuries), treated surgically or conservatively.

## METHODS

*Types of studies included:* This review included randomized or *quasi*-randomized controlled trials.

*Types of participants:* The patients included in this review were 14 years old or over, with soft tissue injuries of the knee (acute or chronic injury of the anterior or posterior cruciate ligament, medial or lateral collateral ligament, menisci, alone or in combination), treated surgically or conservatively.

*Types of interventions:* The rehabilitation protocols that were assessed involved ES as a component of a rehabilitation program after soft tissue injuries of the knee.

*Controls:* The controls received no treatment, placebo, other physical intervention or conventional rehabilitation.

### Types of outcome measurements

*Primary outcomes:* The primary outcomes were muscle strength; functional outcomes (e.g. self-selected walking speed, number of participants using crutches or number of participants who did not progress to the treadmill); pain, as measured using a visual analogue scale; and functional scales, e.g. the activities of daily living (ADL) scale and Lysholm score.

*Secondary outcomes:* The secondary outcomes were range of motion; KT-1000 arthrometer results; subjective and objective laxity/instability of the knee; swelling; and time taken to return to work, at pre-injury level of activity.

### Search strategy to identify studies

We searched the Cochrane Central Register of Controlled Trials (Cochrane Library 2010, Issue 12); Medline (Medical Analysis and Retrieval System Online) via PubMed (1966 to December 2010); Embase (Excerpta Medica Database; 1980 to December 2010); Lilacs (Literatura Latino-Americana e do Caribe em Ciências da Saúde; available at http://bases.bvs.br, from 1982 to December 2010); Cumulative Index to Nursing and Allied Health Literature (CINAHL; 1982 to December 2010); PEDro (Physiotherapy Evidence Database, 1929 to December 2010), at http://www.pedro.fhs.usyd.edu.au/index.html); and the reference lists of studies. We also contacted study authors and experts in order to identify unpublished data. No language restrictions were applied.

The search strategies for randomized controlled trials in PubMed, Embase and Lilacs were combined with subject-specific search terms (electrical stimulation terms and knee injury terms). The strategies of Medline via PubMed were adapted for other databases. The PubMed search strategy was the following: (knee AND (injur* OR ligament* OR tendon* OR (soft tissue) OR menisc*)) AND ((electric* AND stimulation) OR (neuro AND muscle AND stimulation)) AND(randomized controlled trial [pt] OR controlled clinical trial [pt] OR randomized controlled trials [mh] OR random allocation [mh] OR double-blind method [mh] OR single-blind method [mh] OR clinical trial [pt] OR clinical trials [mh] OR ("clinical trial" [tw]) OR ((singl* [tw] OR doubl* [tw] OR trebl* [tw] OR tripl* [tw]) AND (mask* [tw] OR blind* [tw])) OR (placebos [mh] OR placebo* [tw] OR random* [tw] OR research design [mh:noexp] OR comparative study [mh] OR evaluation studies [mh] OR follow-up studies [mh] OR prospective studies [mh] OR control* [tw] OR prospectiv* [tw] OR volunteer* [tw]) NOT (animals [mh] NOT human [mh]).

### Methods

Two authors independently selected trials for inclusion. Any disagreements were resolved through consultation with a third author.

Two authors independently extracted the data. Data relating to methodological issues, participants’ characteristics, interventions and outcome measurements were extracted using a standard extraction form.

Two authors independently assessed trial quality using the Delphi list^18^ (Table 1). The numbers of positive answers to the questions in the questionnaire were expressed as percentages).

**Table 1:** Delphi list^18^

Delphi list	
1. Allocation Was the method of randomization performed? Was the method of random allocation concealed?	Yes/No/Don't know Yes/No/Don't know
2. Were the groups similar at baseline regarding the most important prognostic characteristics?	Yes/No/Don't know
3. Were both inclusion and exclusion criteria specified?	Yes/No/Don't know
4. Was the outcome assessor blinded?	Yes/No/Don't know
5. Was the care provider blinded?	Yes/No/Don't know
6. Was the patient blinded?	Yes/No/Don't know
7. Were point estimates and measures of variability presented for primary outcome measure(s)?	Yes/No/Don't know
8. Did the analysis include an ‘intention-to-treat’ analysis?	Yes/No/Don't know

### Data analysis

For dichotomous data, the relative risk (RR) and 95% confidence interval (CI) were calculated. Continuous outcomes were analyzed using the mean and standard deviation of endpoint measurements, in order to generate the mean difference (MD) and 95% CI. The Rev Man 5 statistical package supplied by the Cochrane Collaboration was used to perform meta-analyses.

The presence of heterogeneity was investigated by means of the chi-square test and the I_2_ test. The heterogeneity was considered statistically significant when I_2_ was greater than 50% and the P-value was less than < 0.10 (< 10%).

## RESULTS

The results from the search were that the following were identified: 177 studies in Medline, of which 21 were selected for evaluation; 145 studies in Embase, of which 18 were selected for evaluation; 51 in PEDro, of which three were selected for evaluation; 33 in the Cochrane Controlled Trials Register, of which 21 were selected for evaluation; and five in Lilacs, of which none were selected. After taking into account the duplicated references in different databases, 19 studies were included and two were excluded.

### Description of the studies included

The sample sizes ranged from eight to 110 patients. Participants’ ages ranged from 14 to 44 years. Most of the studies included (described in Table 2) involved patients undergoing ACL reconstruction. Two involved patients undergoing partial meniscectomy. These studies assessed the effectiveness of ES alongside rehabilitation programs to increase muscle strength, and ES was compared with: conventional exercises without ES and with different frequencies of ES; pulsed electromagnetic stimulation; and biofeedback therapy. The main outcome was muscle strength, which was assessed using an isokinetic dynamometer. Other outcomes assessed included: knee function questionnaires, KT-1000 arthrometer results, number of participants using crutches, number of participants whose rehabilitation did not progress to the use of a treadmill, walking velocity and stand time for the leg involved. Outcome measurements (isokinetic evaluation) were made between 1 and 52 weeks after surgery, and the isokinetic protocol involved differences in muscle contraction (isometric or isokinetic), test velocity and knee isometric test angle.

**Table 2:** List of studies about electrical stimulation (ES) included in this review

Anderson et al.^28^ Setting: Nashville, Tennessee, USA	Inclusion criteria: patients with cruciate ruptures after surgery Age (mean) and sex: – group 1: 20 years, 17 male and 2 female – group 2: 23 years, 10 males and 8 females – group 3: 20.4 years, 11 males and 9 female – group 4: 19.8 years, 16 males and 4 female – group 5: 22.8 years, 11 males and 8 females	Group 1: knee immobilizer in extension + quadriceps exercise Group 2: knee immobilizer in extension + quadriceps exercise + TENS Group 3: knee immobilizer in 60º flexion+ quadriceps exercise + TENS Group 4: knee immobilizer in 60º flexion+ quadriceps exercise + ES 10 hrs. a day Group 5: knee immobilizer in 60º flexion+ quadriceps exercise + TENS + CPM (35-70º) ES parameters: F: 35 Hz, pulse width of 150 microsec. Time on: 10 sec and time off: 110 sec, for 3 months. Intensity: 65 to 100 mA
Buhmann et al.^26^ Setting: Göttingen, Germany	Inclusion criteria: patients with cruciate ruptures after surgery Age: – group 1: 18-47 years – group 2: 21-43 years – group 3: 20-44 years	Group 1: functional aftercare immediately after surgery consisting of therapeutic exercises and physiotherapy Group 2: ES from day 7 after surgery, additionally to the therapeutic exercises and physiotherapy Group 3: as group 2 and additionally, isokinetic training from week 9 onwards after surgery. ES parameters: – F: 50 Hz, time on: 10 sec and time off: 20 sec, 30 minutes Isokinetic training parameters: 10 contractions at 60º/s, 120º/s and 180 º/s
Currier et al.^33^ Setting: University of Kentucky, USA	Sample: 17 Inclusion criteria: anterior cruciate ligament reconstruction, age ranging from 15 to 39 years	Sequential allocation: NMES or NMES and PEMF groups. Standard ACL protocol that included range of motion, muscle setting, straight leg raise, and ambulation exercises during the first 6 weeks after surgery. NMES: 2500 Hz delivered in 50 bursts per second with a 10-ms "on" time and a 10-ms "off’ time. Each induced contraction lasted 15 seconds (5-sec ramp on), followed by 50 seconds off for 10 n contractions per session. The NMES and PEMF was administered with physical therapy in the fourth and each succeeding session as outpatient treatment, three times per week for 5 weeks (total = 6 weeks, 18 NMES and NMES/ PEMF treatments, two assessments).
Delitto et al.^23^ Setting: Washington University Medical School, USA	Inclusion criteria: anterior cruciate ligament reconstruction and age between 19 to 44 years. Sample: 20	ES (n = 10): – F: 2500 Hz/50 Hz, 5 days a week, for 3 weeks. – Repetitions: 15, time on: 15 sec, time off: 50 sec. Control Group (n = 10): conventional rehabilitation. Intervention: 6 weeks after surgery
Draper and Ballard^32^ Setting: Knoxville Orthopedic Clinic, USA	Inclusion criteria: between 15 and 44 years. ACL reconstruction. Exclusion criteria: collateral ligament, posterior cruciate ligament injury or osteotomy. Sample: 30	ES (n = 15): F: 35 Hz, time on: 10 sec, time off: 20 sec. Biofeedback (n = 15)
Eriksson and Häggmark^28^ Setting: Stockholm, Sweden	Inclusion criteria: patients with cruciate ruptures after surgery, with age between 20 and 40 years	All patients underwent isometric quadriceps training. Group 1 (n = 4): ES, F: 200 Hz, time on: 5-6 sec and time off: 5 sec, for 1 hr, 5 days a week for 4 weeks Group 2 (n = 4): conventional protocol
Fitzgerald et al.^15^ Setting: USA	Inclusion criteria: 14 years and above. Exclusion criteria: rehabilitation in another setting. Sample size: 31, underwent anterior cruciate ligament reconstruction.	1. ES group (n = 17) F: 2500 Hz, pulsed at 75 Hz, time on: 10 sec, time off: 50 sec, 2 days a week 10 repetitions. Total time: 11-12 min. 2. Control group (n = 14) Conventional Rehabilitation.
Lainey et al.^35^ Setting: General and Marine Hospital, Owen Sound, Canada	Inclusion criteria: patients underwent meniscectomy surgery	Group 1: exercise alone followed by exercise plus stimulation, then they reverted to exercise alone, finishing with exercise plus stimulation. Group 2: performed the reverse of the training of group 1. They trained five times a week for the first two weeks and three times a week for the following four weeks. ES characteristics: 4000 Hz, pulsed at 100 Hz, four electrodes.
Lieber et al.^25^ Setting: San Diego, California, USA	Inclusion criteria: surgical reconstruction of the anterior cruciate ligament within previous 2-6 weeks and the ability to position the knee in 90º flexion. Sample: 40 men and women, 15-44 years	Group 1: exercise Group 2: exercise + ES: 10 sec on and 20 sec off, Frequency: 50 Hz pulse duration: 250 microsec Duration: 4 weeks All the subjects were allowed to participate in a home program
Paternostro-Sluga et al.^21^ Setting: Austria	Inclusion criteria: Anterior cruciate ligament reconstruction Sample: 49	1. ES + exercises Program 1: F: 30 Hz, time on: 5 sec, time off: 15 sec, 12 repetitions. Program 2: F: 50 Hz, time on: 10 sec, time off: 50 sec, 12 repetitions. 2. TENS + Exercises F: 100 Hz, 30 minutes, sensitive threshold. Six weeks of intervention. One control group (exercises).
Rebai et al.^13^ Setting: France	Inclusion criteria: sportsmen, aged between 22 and 35 years, ACL reconstruction. Exclusion criteria: collateral ligament or meniscal injuries. Sample: 8	1. ES group: - F: 20 Hz, time on: 15 sec, time off: 10 sec, total time: 60 min. 2. ES (different frequency): F: 80 Hz, time on: 15 sec, time off: 75 sec, total time: 60 min.
Ross et al.^29^ Setting: Ohio, USA	Inclusion criteria: underwent ACL reconstruction Age: 27.1+ 4.89 (CK chain) and 28.4+5.91 exclusion criteria: meniscal and ligament injuries associated	All patients performed isometric quadriceps training for 1 week postoperatively. CK chain group: Rehabilitation with CK chain exercises ES group: F: 50 Hz, time on: 15 s, time off: 35 s, for 6 weeks Group 2: instruction in isometric co-contraction of the thigh muscles Duration: 6 postoperative weeks
Siebert et al.^31^ Setting: USA	Inclusion criteria: underwent ACL reconstruction. Sample: 52 (34 men and 18 women) Age: 27.4	Group 1: Daily standardized postoperative exercise program. Group 2: ES: 25 Hz or 1100 Hz 6 times a day, 30 min. CT scan Assessment: Before operation and 2 weeks, 6 weeks and 12 weeks afterwards.
Sisk et al.^14^ Setting: USA	Inclusion criteria: ACL reconstruction Sample: 23 Age: ES (23.4 ± 7.5) and non-ES: (23.9 ± 9.2) years	ES group: 6 weeks, F: 40 Hz, T on: 10 sec, T off: 30 sec. Exercise group: conventional rehabilitation.
Staub et al.^27^ Setting: Erfurt, Germany	Inclusion Criteria: ACL reconstruction Sample: 93	ES group: 4 weeks, F: 50 Hz, T on: 110 sec, T off: 20 sec. Exercise group: conventional rehabilitation.
Snyder-Mackler et al.^16^ Setting: Boston, USA	Inclusion criteria: ACL reconstruction Sample: 10, age: 18 to 28 years	ES group: F: 2500 Hz, T on: 15 sec., T off: 50 sec. Exercise group: conventional rehabilitation. Duration of intervention: 6 weeks.
Snyder-Mackler et al.^24^ Setting: Delaware, Pittsburgh, St. Louis, Germantown, USA	Inclusion criteria: age: 15 to 43 years; diagnosis: ACL reconstruction with Achilles tendon graft (n = 18), patellar-ligament allograft (n = 10), semitendinosus and gracilis tendons (n = 7), autologous patellar-ligament (n = 75). Exclusion criteria: none reported	All patients were managed three times a week, from the first to sixth postoperative week. Group 1: High-intensity ES: f: 2500 Hz at a burst rate of 75 bursts per second, Time on: 11 s, Time off: 120 s, number of contractions: 15 Group 2: high volitional exercise, three times a week, number of contractions: 15 positioned sitting with the knee in 60º of flexion on isokinetic dynamometer. Group 3: low-intensity ES four times a day, five days a week Time on: 15 s, time off: 50 s, frequency: 55 Hz Group 4: The patients received the treatment of groups 1 and 3. They did a combination of high and low-intensity ES.
Wigerstad-Lossing et al.^22^ Setting: Sweden	Inclusion criteria: ACL reconstruction. Sample: 23 Age: 18 to 28 years.	Exercise group: ankle exercises, quadriceps isometric contractions, hip abduction. ES group: F: 30 Hz., T on: 6 sec, T off: 10 sec.
Williams et al.^34^ Setting: California, USA	Inclusion criteria: post-arthroscopic meniscectomy patients Sex: 3 females and 18 males Age: range 18 to 45 years, mean 33 years	ES group: ES to the quadriceps 10 minutes, 5 times a week, frequency: 2500 Hz, pulsed at 50 Hz, 15 sec of contraction and 50 sec of resting. Control group: quadriceps and hamstring isometrics and an isotonic progressive resistance program 3 times a week.

ACL = anterior cruciate ligament; TENS = transcutaneal electrical nerve stimulation; NMES = neuromuscular electrical stimulation; PEMF = pulsed electromagnetic field; CK = closed kinetic.

### Excluded studies

The studies that were excluded^19,20^ are presented in Table 3.

**Table 3:** Excluded studies

Franke et al.^19^	Retrospective study
Hörster et al.^20^	Only electromyography (EMG) assessment

### Methodological quality of the studies included (Table 4)

**Table 4:** Methodological quality of studies included, using Delphi list criteria

Study/Delphi list	1a	1b	2	3	4	5	6	7	8	% yes
Anderson and Lipscomb^28^	Yes	Don't know	Yes	Yes	Don't know	No	No	No	No	33.3%
Buhmann et al.^26^	Yes	Don't know	Yes	Yes	Don't know	No	No	Yes	No	44.4%
Currier et al.^33^	Yes	Don't know	Yes	Yes	Don't know	No	No	Yes	No	44.4%
Delitto et al.^23^	Yes	Don't know	Yes	Yes	Yes	No	No	No	No	44.4%
Draper and Ballard^32^	Yes	Don't know	Yes	Yes	Don't know	No	No	Yes	No	44.4%
Eriksson and Häggmark^30^	Yes	Don't know	Yes	Yes	Don't know	No	No	No	No	33.3%
Fitzgerald et al.^15^	Yes	Don't know	Yes	Yes	Yes	No	No	Yes	Yes	66.6%
Lainey et al.^35^	Yes	Don't know	Yes	Yes	Don't know	No	No	No	No	33.3%
Lieber et al.^25^	Yes	Don't know	Yes	Yes	Don't know	No	No	No	No	33.3%
Paternostro-Sluga^21^	Yes	Don't know	Yes	Yes	Yes	No	No	Yes	No	55.5%
Rebai et al.^13^	Yes	Don't know	Yes	Yes	Don't know	No	No	No	No	33.3%
Ross et al.^29^	Yes	Don't know	Yes	Yes	Don't know	No	No	Yes	No	44.4%
Siebert et al.^31^ (congress abstract: not enough data to be evaluated)										
Sisk^14^	Yes	Don't know	Yes	Yes	Don't know	No	No	Yes	No	44.4%
Snyder-Mackler et al.^16^	Yes	Don't know	Yes	Yes	Yes	No	No	Yes	No	55.5%
Snyder-Mackler et al.^24^	Yes	Don't know	Yes	Yes	Yes	No	No	Yes	No	55.5%
Staub et al.^27^	Yes	Don't know	Yes	Yes	Yes	No	No	No	No	44.4%
Wigerstad-Lossing et al.^22^	Yes	Don't know	Yes	N	Don't know	No	No	Yes	No	33.3%
Williams et al.^34^	Yes	Don't know	Yes	Yes	Don't know	No	No	Yes	No	44.4%

The studies that were included presented some methodological flaws. None of the studies described concealment of allocation at the time of randomization. Among the studies with drop outs,^13-15,21,22^ only Fitzgerald et al.^15^ mentioned intention-to-treat analysis. The baseline participant characteristics were well described and the groups were comparable. The interventions and outcomes were well defined and described. Blinded outcome assessment was reported in four studies.^15,21,23,24^ None of the studies included reported sample size calculations.

### Results from comparisons

#### Comparison 1: conventional rehabilitation with and without ES

##### Isometric quadriceps peak torque

**Figure 1:**
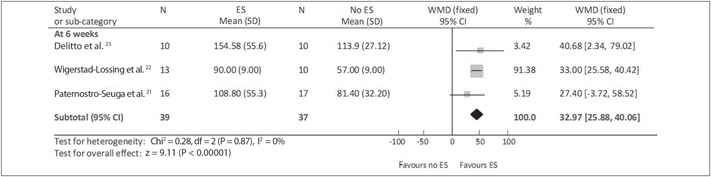
Meta-analysis graph

At four weeks after surgery, according to Lieber et al.,^25^ the magnitude of the increase in isometric quadriceps torque was not significantly different between the groups. At six, nine and twelve weeks after the operation, the decrease in isometric quadriceps torque in the stimulation group during immobilization was significantly less than that of the group without stimulation (without correlatable data). At six weeks after surgery, Delitto et al.,^23^ Paternostro-Sluga et al.^21^ and Wigerstad-Lossing et al.^22^ assessed the isometric quadriceps peak torque. There was a statistically significant difference in favor of ES (MD −32.70 Nm; 95% CI: −39.92 to −25.48) (Figure 1).

Sisk et al.^14^ assessed the isometric quadriceps peak torque with the knee at seven weeks (MD 0.03 Nm; 95% CI: −0.29 to 0.35), eight weeks (MD −0.07 Nm; 95% CI: −0.42 to 0.28) and nine weeks (MD −0.02 Nm; 95% CI: −0.46 to 0.42) after surgery. There were no statistically significant differences between the times assessed. It was not possible to pool the data because of the different dates of isokinetic evaluation. Nine weeks after surgery, the group with ES additionally showed significantly better results for the maximum strength transmitted via the isokinetic system.^26^

Paternostro-Sluga et al.^21^ assessed the quadriceps isometric peak torque at 12 weeks (MD −14.2 Nm; 95% CI: −16.55 to 44.95) and 52 weeks (MD −20.5 Nm; 95% CI: −5.49 to 46.49) after surgery. There was no statistically significant difference between the groups with and without ES. After one year, Lieber et al.^25^ did not find any significant difference between the groups.

##### Isokinetic quadriceps peak torque

At six and eight weeks after surgery, the isokinetic peak torque was assessed at test velocities of 60 º/s^19^ and 90 º/s and 210 º/s.^16^ There was a statistically significant difference in favor of ES at test velocities of 90 º/s (MD −51.20 Nm; 95% CI: −72.1 to −30.3) and of 210 º/s (MD −38.8 Nm; 95% CI: −54.9 to −22.7), but not at a test velocity of 60 º/s. At 12 weeks (MD −51.00 Nm; 95% CI: −51 to −10.2) and 52 weeks (MD −26.80 Nm; 95% CI: −54.51 to 0.91) after surgery, Paternostro-Sluga et al.^21^ found that there was no statistical significant difference in quadriceps isokinetic peak torque between the groups. It was not possible to pool the data for this outcome because of the different isokinetic test velocities used and the different assessment times. Buhmann et al.^26^ found that the greatest increase in the group that received ES occurred over the final four weeks of the study. Staub et al.^27^ did not find any statistical significance between the ES group and the control group regarding increases and decreases in isometric peak torque at either 12 weeks or nine months after the operation. At 18 weeks, Anderson and Lipscomb^28^ found a statistically significant difference favoring the ES group.

##### Isometric quadriceps index

Two studies^15,23^ assessed the effectiveness of ES using the isometric quadriceps index at six, 12 and 16 weeks after surgery. There was a statistically significant difference in favor of ES six weeks after surgery (MD −27.10%; 95% CI: −39.37 to −14.83), whereas there was no statistically significant difference at 12 weeks (MD −8.9 Nm; 95% CI: −19.89 to 2.09) and 16 weeks (MD −8.10 Nm; 95% CI: −18.09 to 1.89) after surgery. It was not possible to do a meta-analysis because of the different evaluation dates. Snyder-Mackler et al.^24^ found at six weeks after surgery that the quadriceps had achieved 70% recovery with high-intensity exercise and 57% with volitional exercise.

##### Isokinetic quadriceps index

At eight weeks after surgery, Snyder-Mackler et al.^16^ assessed the isokinetic quadriceps index at two test velocities. There was a statistically significant difference in favor of ES at the test velocities of 90 º/s (MD −23.4%; 95% CI: −29.32 to −17.48) and 210 º/s (MD −25.2 Nm; 95% CI: −30.07 to − 20.33).

#### Functional outcomes

##### ADL questionnaire

At 12 and 16 weeks after surgery, Fitzgerald et al.^15^ showed that there was a statistically significant difference in favor of ES in relation to the ADL questionnaire on both occasions (MD −7 Nm; 95% CI: −12.78 to −1.22; and MD −5.1 Nm; 95% CI: −9.74 to −0.46).

##### Lysholm score (without correlatable data)

At 12 weeks and nine months, there was no statistical significance between the ES and control group regarding Lysholm and Tegner scores in the study by Staub et al.^27^

##### Range of motion

At 18 weeks, Anderson and Lipscomb^28^ found a greater range of motion in the ES group (without correlatable data).

##### Number of participants using crutches

At four and eight weeks after surgery, Fitzgerald et al.^15^ found that there were no statistically significant differences in the number of participants using crutches.

##### Number of participants who did not progress to treadmill running

Fitzgerald et al.^15^ assessed the number of participants who did not progress to treadmill running and found that there were no statistically significant differences at 12 weeks (RR 0.7; 95% CI: 0.36 to 1.36) or 16 weeks (RR 0.45; 95% CI: 0.13 to 1.51) after surgery.

##### Gait analysis

Gait was assessed in one study.^16^ There were statistically significant differences in favor of ES regarding gait velocity (MD −0.28 m/s; 95% CI: −0.34 to −0.22), standing time on the side involved (MD −6.80 s; 95% CI: −8.92 to −4.68) and cadence (MD −6.10; 95% CI: −8.22 to −3.98).

##### Fastex test (gait step test, stabilization step test and quick feet test)

At 12 weeks and nine months after surgery, Staub et al.^27^ did not find any statistically significant difference between the ES and control group (without correlatable data) in the Fastex test.

##### Unilateral squat test, lateral step test and forward reach test

At six weeks after the operation, Ross^29^ did not find any statistical significance between the groups in the unilateral squat test (MD 6.07; 95% CI: −8.81 to 20.95), lateral step test (MD −3.30 s; 95% CI: −1.20 to 7.80) or forward reach test (MD 58.75 s; 95% CI: 0.45 to 5.90).

#### Knee pain

Fitzgerald et al.^15^ found that there were no statistically significant differences in knee pain at 12 weeks (MD −0.3; 95% CI: −0.7 to 1.3) or 16 weeks (MD −0.2; 95% CI: −0.89 to 0.49) after the surgery.

#### Muscle strength (manual muscle test)

Patients who underwent ES performed better in the manual muscle test than did the control group.^30^

#### KT-1000 (instrumental stability test)

Buhmann et al.^26^ did not find any significant differences between the treatment groups (no data presented).

#### Computed tomography scan

Siebert et al.^31^ studied patients who underwent knee ligament surgery. At six weeks after the operation, the quadriceps area had become significantly reduced in the control and physical exercise group, in comparison with the group that had exercise combined with electrical stimulation.

#### Comparison 2: Conventional rehabilitation with ES versus conventional rehabilitation with biofeedback

One study made this comparison. At six weeks after surgery, Draper and Ballard^32^ found a statistically significant difference in favor of biofeedback regarding the isometric quadriceps index (MD −8.5 Nm; 95% CI: −16.72 to −0.28).

#### Range of motion

There was no difference in the range of motion between the two groups at one week (MD 0.4; 95% CI: −2.12 to 2.92), two weeks (MD −0.6; 95% CI: −4.43 to 3.23) or four weeks (MD 1.0; 95% CI: −2.55 to 4.55) after surgery.^32^

#### Comparison 3: Conventional rehabilitation with ES of 20 Hz versus conventional rehabilitation with ES of 80 Hz

##### Isometric quadriceps peak torque

At 12 weeks after surgery, Rebai et al.^13^ found that there was no statistically significant difference between using ES of 20 Hz and using ES of 80 Hz. After 12 weeks of rehabilitation, the quadriceps peak torque deficit was less than 30%, except for two patients in the 20 Hz stimulated group (without correlatable data).

##### Volume deficit

The thigh muscle volume deficit in the operated limb was between 3% and 9% in the 20 Hz stimulated group and between 1% and 2% in the 80 Hz stimulated group, in the study by Rebai et al.^13^ (without correlatable data).

#### Comparison 4: Conventional rehabilitation with high-intensity ES of 2500 Hz at a burst rate of 75 Hz versus conventional rehabilitation with low-intensity ES of 55 Hz

##### Isometric quadriceps index

Snyder-Mackler et al.^24^ found at six weeks after surgery that the degree of quadriceps recovery was 70% with high-intensity training and 51% with low-intensity training (without correlatable data).

#### Comparison 5: ES versus ES plus pulsed electromagnetic field

##### Thigh girth

In the study by Currier et al.,^33^ at six weeks after surgery, there was no difference between the groups regarding thigh girth measured at 12 cm (MD −2.8 cm; 95% CI: −8.17 to 2.57), 20 cm (MD −1.6 cm; 95% CI: −7.38 to 4.18) and 25 cm (MD −1.4 cm; 95% CI: −7.21 to 4.41) proximally to the superior patellar pole.

### Results for meniscectomy patients

#### Comparison: Conventional rehabilitation with ES versus conventional rehabilitation

##### Isometric quadriceps peak torque

There was no significant difference between the groups in the study by Williams et al.,^34^ at three weeks after meniscectomy (MD 22.3 Nm; 95% CI: −0.1 to 4.47). At six weeks, no differences were found between the groups in the study by Lainey et al.^35^ (without correlatable data).

## DISCUSSION

The studies included did not address all of our objectives. Heterogeneous strength assessment protocols (evaluation data, test position and test speed) need to be standardized in order to compare results from ES studies. The participants in the studies included in this review were patients who underwent surgical reconstruction of the anterior cruciate ligament and meniscectomy, so the results from this review should not be generalized to people with collateral or posterior cruciate ligament injuries.

This review found that muscle strength was higher six weeks after surgery with ES. This finding can be explained by two theories: firstly, a combination of ES and voluntary exercise results in more exercise for the muscle; and secondly, there is preferential activation of type II fibers through ES. Activation of both types of fibers may result in an improvement of strength. Better results from ES over the initial rehabilitation period may be associated with weaker muscles over this period (six to eight weeks after surgery), and an additional stimulus (ES) makes a strong difference. We recommend that ES should be used over the first six weeks after surgery. Based on the studies included, we recommend that the ideal low frequency for increasing muscle strength ranges from 35 to 80 Hz. For cases of medium frequency, the modulation should be at 2500 Hz/50-75 Hz. The pulse duration should be around 200 to 350 microseconds and it is recommended that the relationship between contraction and resting times should be about 1:5 during the early rehabilitation phase.

Functional assessment is extremely important because returning to normal activities is the main goal of rehabilitation. Functional improvements were achieved through the use of ES, in relation to the following outcomes: activities of daily living scale, walking velocity and time spent standing on the injured leg.

One possible limitation of our study is that we carried out just one meta-analysis with only three studies, because of data heterogeneity. For the studies without correlatable data, we made a description of the results.

We carried out a literature search in which we placed importance on the quality aspects of the studies. The Consolidated Standards for Reporting Trials (CONSORT) Statement is a checklist of items that the reports on clinical trials must follow. The CONSORT Statement,^36^ which was created in 1999 and updated in 2010, aims to improve reports on randomized clinical trials, help authors to recognize suitable study designs and assess study validity. Most of the studies included in this review did not follow the precepts of the CONSORT Statement: there was no reporting of allocation concealment in any study; none of them reported sample size calculation; and, of the studies with drop-outs, only one mentioned intention-to-treat analysis. Using the Delphi list to assess the studies, the studies were found to fulfill at least half of the items: the groups of patients were similar at the baseline; all the inclusion criteria were well described; all the studies were randomized; and, in three studies, the assessor was blinded.

A systematic review published in 2005^37^ assessed the effectiveness of ES on the quadriceps muscles of both healthy and injured individuals. The present study focused only on injured individuals and, in addition to strength, we reported knee function outcomes. In contrast to Bax et al.,^37^ we included only randomized controlled clinical trials. One of the conclusions of the previous systematic review was that ES might be more effective than voluntary exercises on weakened muscles. Our review agrees with another systematic review published in 2010,^38^ which included eight studies and concluded that ES combined with exercise might be more effective in improving quadriceps strength than exercise alone and that inconsistencies were noted in the ES parameters and their application.

## CONCLUSION

The evidence available from randomized clinical trials of limited quality shows that electrical stimulation, in combination with a conventional rehabilitation program, might be more effective for improving muscle strength and function for up to two months after ACL reconstruction than conventional rehabilitation alone.

### Implications for research

Randomized controlled trials of better methodological quality, with adequate sample size and with at least 12 months of follow-up are necessary to ascertain the effectiveness of ES for increasing the muscle strength of patients with soft tissue injuries of the knee.
